# HIV infected pregnant women monitored in the Adults 3 Department of the National Institute for Infectious Diseases “Prof. Dr. Matei Balş” – risk of mother to child transmission

**DOI:** 10.1186/1471-2334-13-S1-P26

**Published:** 2013-12-16

**Authors:** Cristina Popescu, Mariana Mărdarescu, Raluca Dulama, Raluca Mihăilescu, Daniela Munteanu, Ruxandra Moroti, Violeta Molagic, Adriana Hristea, Mihaela Rădulescu, Cătălin Tilişcan, Raluca Năstase, Iulia Niculescu, Alina Lobodan, Anca-Ruxandra Negru, Irina Lăpădat, Ligia Ionescu, Victoria Arama

**Affiliations:** 1 National Institute for Infectious Diseases “Prof. Dr. Matei Balş”, Bucharest, Romania; 2 Carol Davila University of Medicine and Pharmacy, Bucharest, Romania

## Background

It was estimated that at the end of 2010 more than 34 million people are infected with HIV worldwide and 50% of them are women. After 1989 in Romania, mother-to-child transmission of HIV remains the primary way of child infection with HIV. We analyzed mother to child transmission risk factors between 2005 and 2013.

## Methods

We analyzed 18 mother-infant pairs in order to establish the risk factors for HIV transmission. We performed a retrospective study of the HIV infected women monitored in the Adults 3 Department of the National Institute for Infectious Diseases “Prof. Dr. Matei Balş”.

## Results

Only 8 of the pregnant women discovered the HIV infection before childbirth and received antiviral prophylaxis: 6 with combivir and lopinavir/r, 1 with combivir and atazanavir/r and 1 with combivir and viramune. All of these patients had caesarean delivery and formula feeding for the child. All new-born were HIV negative. 10 women were diagnosed with HIV infection after the delivery: 9 had a vaginal delivery and one caesarean delivery. None of the women took chemoprophylaxis and all patients breastfed the children. 2 children were HIV positive. Both HIV positive children were born from mothers with high viral loads and low CD4 counts.

## Conclusion

All pregnant women must be HIV-tested before delivery in order to reduce the risk of mother-to-child transmission of HIV.

